# Application of Methods Detecting Xenotransplantation-Relevant Viruses for Screening German Slaughterhouse Pigs

**DOI:** 10.3390/v16071119

**Published:** 2024-07-11

**Authors:** Hina Jhelum, Benedikt Kaufer, Joachim Denner

**Affiliations:** Institute of Virology, Free University Berlin, 14163 Berlin, Germany; hina.jhelum@fu-berlin.de (H.J.); benedikt.kaufer@fu-berlin.de (B.K.)

**Keywords:** porcine viruses, xenotransplantation, virus safety, herpes viruses, porcine endogenous retroviruses, latency

## Abstract

Detection methods have been developed to prevent transmission of zoonotic or xenozoonotic porcine viruses after transplantation of pig organs or cells to the recipient (xenotransplantation). Eleven xenotransplantation-relevant viruses, including porcine cytomegalovirus, porcine roseolovirus (PCMV/PRV), porcine lymphotropic herpesviruses -1, -2, -3 (PLHV-1, 2, 3), porcine parvovirus (PPV), porcine circovirus 2, 3, 4 (PCV2, 3, 4), hepatitis E virus genotype 3 (HEV3), porcine endogenous retrovirus-C (PERV-C), and recombinant PERV-A/C have been selected. In the past, several pig breeds, minipigs, and genetically modified pigs generated for xenotransplantation had been analyzed using these methods. Here, spleen, liver, and blood samples from 10 German slaughterhouse pigs were screened using both PCR-based and immunological assays. Five viruses: PCMV/PRV, PLHV-1, PLHV-3, and PERV-C, were found in all animals, and PCV3 in one animal. Some animals were latently infected with PCMV/PRV, as only virus-specific antibodies were detected. Others were also PCR positive in the spleen and/or liver, indicative of an ongoing infection. These results provide important information on the viruses that infect German slaughterhouse pigs, and together with the results of previous studies, they reveal that the methods and test strategies efficiently work under field conditions.

## 1. Introduction

Xenotransplantation is under development to alleviate the shortage of human organs for transplantation [1]. Pigs are the species of choice, and multiple genetically modified pigs have been generated to prevent hyperacute and acute rejection of pig organs [2]. In preclinical studies with non-human primates using organs from such pigs and novel immunosuppressive drugs, considerable survival times for the xenotransplant were achieved in the last several years. Based on these studies, a heart from a pig with 10 genetic modifications (10 GE pigs) was transplanted into a patient at the University of Maryland in Baltimore (UMB) in 2022. The patient survived for 2 months [3]. In the year before, surgeons from the New York University (NYU) Langone Transplant Institute xenotransplanted α GalT-KO pig thymo-kidneys into a brain-dead human recipient, which lasted for 54 h [4,5]. In a study at the University of Alabama in Birmingham (UAB), decedent recipients were nephrectomised and bilaterally transplanted with kidneys from 10 GE pigs. Patients received conventional immunosuppression, and the first case was monitored for 74 h, during which urine production was poor and creatinine clearance did not improve [6]. Furthermore, at NYU, 10 GE pig hearts were transplanted into brain-dead patients [7]. In China, a 50-year-old clinically dead man was the first person to receive a liver from a pig [8]. Also in China, at the Xijing Hospital of the Air Force Medical University in Xian, the kidney of a multi-gene-edited pig was transplanted into a brain-dead human. The kidney was functioning well for more than 13 days [9]. At the UMB, a second heart was transplanted into a patient, who died after 6 weeks [10]. Recently, pig kidneys with 69 genetic modifications were transplanted at the Massachusetts General Hospital in Boston. The patient was in excellent health and had been discharged from hospital [11]. He died after 7 weeks from a heart attack; the kidneys worked well at that time. At the NYU Langone Transplant Institute, a left ventricular assist device and a pig kidney with only one genetic modification, a knockout of the gene responsible for the production of alpha-gal residues, were transplanted in a living person [12].

Xenotransplantation could result in the transmission of potentially pathogenic microorganisms to the recipient. The fact that the porcine cytomegalovirus, a porcine roseolovirus (PCMV/PRV), was transmitted to the pig heart recipient in Baltimore [3], and that the virus obviously contributed to the death of the patient, demonstrates how important it is to prevent virus transmission [13]. In the past, it was shown in several studies that transmission of PCMV/PRV with pig heart and kidney transplants into non-human primates (NHP) resulted in a significant reduction in the survival time of the xenotransplant [14,15,16,17]. It is important to be aware that microorganisms, including viruses such as the human immunodeficiency virus, human cytomegalovirus, and rabies virus, have also been transmitted in allotransplantations [18].

The data mentioned above underline the importance of safety research in this field. Consequently, in the last few years, numerous PCR-based and immunological methods have been developed to detect porcine viruses [19,20,21,22]. At present, it is still unclear which viruses may pose a risk during xenotransplantation, i.e., which viruses are xenotransplantation relevant [23,24]. On one hand, there are known zoonotic viruses (zoonosis means inducing disease in the recipient), such as the hepatitis E virus, genotype 3 (HEV3). On the other hand, we now know viruses that do not harm healthy people but harm patients in the context of xenotransplantation, such as PCMV/PRV. These viruses should be called xenozoonotic [25]. Unfortunately, tests for these pig viruses are not available at most veterinary diagnostic institutes, but only in very few specialized laboratories [26].

Altogether, assays for more than 11 viruses were developed [21]. In addition to HEV3 and PCMV/PRV, discussed above, porcine circovirus type 2 (PCV2), PCV3, and PCV4 were included. PCV2 induces an entire complex of diseases called PCV2-related disease (PCVD) (for review, see [27]). PCV3 is also pathogenic in pigs, whereas PCV4 is new, was for the first time detected in China, and was recently also found in wild boars and commercial pigs in Europe [28]. Porcine endogenous retroviruses (PERVs) were included because they are integrated in the genome of all pigs and may infect human cells. Retroviruses are known to induce immunodeficiencies and/or tumors, and the closest relatives of PERV, murine leukemia viruses, feline leukemia viruses, and koala retroviruses have been shown to induce immunodeficiencies or tumors in the infected hosts [29]. The other viruses were selected based on the recommendations of the Federation of European Laboratory Animal Science Associations (FELASA) [30], the list of viruses tested by Ellegaard Göttingen Minipigs A/S, Dalmose, Denmark, providing Göttingen Minipigs for biomedical research wordwide [31], and the list of viruses declared by Fishman as “not permitted in swine with designated pathogen-free status” [13].

To test the developed methods under field conditions, we have used them in the past for screening not only pigs generated for xenotransplantation and the corresponding non-human primate recipients [16,32], but also for a comprehensive screening of pig breeds such as the indigenous Greek black pigs [33], the Göttingen minipigs [24,34,35,36], the Aachen minipigs [37], the Mini LEWE minipigs [38], Göttingen minipigs with dippity pig syndrome [39], and Greek pigs with erythema multiforme [40]. Here, we screen German slaughterhouse pigs using PCR-based and immunological methods able to detect pig viruses and demonstrate again the robust functionality of these methods. Furthermore, we compare the data to previously tested pig breeds.

## 2. Materials and Methods

### 2.1. Animals and Tissues

Tissue samples from spleen, liver, and clotted blood were obtained from pigs presenting a breeding between Duroc-boars Danish landrace x Danish Large White (Edelschwein) hybrid sows from a slaughterhouse near Berlin; their age was 6 months. Sex and weight are shown in Table 1. The samples were frozen at −80 °C until analyzed.

### 2.2. Isolation of DNA and RNA

DNA and RNA were isolated from the tissues and purified PBMCs according to the manufacturer’s instructions using the DNeasy Blood and Tissue Kit as well as the RNeasy Kit (Qiagen, Hilden, Germany). DNA and RNA concentrations were determined using a NanoDrop ND-1000 (Thermo Fisher Scientific Inc., Worcester, MA, USA) or Qubit device (Invitrogen, Thermo Fisher Scientific Inc., Worcester, MA, USA).

### 2.3. Real-Time PCR for the Detection of DNA Viruses

Real-time PCRs using specific primers and probes were performed with a defined sensitivity to detect PCMV/PRV (sensitivity 10 copies/100 ng DNA), PLHV-1 (1 copy/100 ng DNA), PLHV-2 (1 copy/100 ng DNA), PLHV-3 (1 copy/100 ng DNA), PCV2 (1 copy/100 ng DNA), PCV3 (10 copies/100 ng DNA), PCV4 (100 copies/100 ng DNA), and PPV1 (10 copies/100 ng DNA) as described previously [33]. The primers and probes are listed in Table 2. All protocols were performed using the SensiFAST Probe No-ROX Kit (Meridian Bioscience, Cincinnati, OH, USA) in a reaction volume of 16 µL plus 4 µL (100 ng) of DNA template. Duplex real-time PCRs were performed, testing simultaneously the viral gene of interest and porcine glyceraldehyde-3-phosphate-dehydrogenase (pGAPDH) as an internal control. The functionality of the PCRs was verified using virus-specific gene blocks containing the sequence of the primers and the probe [38]. Real-time PCR reactions were carried out using a qTOWER3 G qPCR cycler (Analytik Jena, Jena, Germany) and the real-time PCR conditions as previously described [33].

### 2.4. Real-Time Reverse Transcriptase PCR for the Detection of HEV3

To detect hepatitis E virus genotype 3 (HEV3), a real-time reverse transcriptase-PCR (real-time RT-PCR) was carried out. This method was first described by Jothikumar et al. [41]. The reactions were performed in a reaction volume of 16 µL using the SensiFAST Probe No-ROX One-Step Kit (Meridian Bioscience, Cincinnati, OH, USA) plus 4 µL (100 ng) of template RNA. A reaction without reverse transcriptase was performed to demonstrate the absence of DNA contamination. A qTOWER^3^ G PCR cycler (Analytik Jena, Jena, Germany) was used and A reverse transcriptase step of 30 min at 50 °C, was followed by an activation step of 15 min at 95 °C and 45 cycles comprising a step of 10 s at 95 °C, followed by a step of 20 s at 55 °C and 15 s at 72 °C.

### 2.5. Conventional and Real-Time PCR for the Detection of PERVs

To determine the presence of PERV-C, a conventional PCR (described as PCR4 in [49]) was performed using a set of primers, which resulted in an amplicon of a length of 288 bp. AmpliTaq DNA Polymerase (Applied Biosystems, Waltham, MA, USA) was used, and the reaction was set up in a Biometra TRIO cycler (Analytik Jena, Jena, Germany). An activation step of 95 °C for 10 min was followed by 45 cycles composed of denaturation steps (95 °C for 15 s), annealing steps (55 °C for 30 s), extension steps (72 °C for 30 s), and a final extension at 72 °C for 5 min.

To determine the presence of human-tropic PERV-A/C, a conventional PCR was set up using specific primer pairs (Table 2), which produces an amplicon of 1266 bp length [50]. AmpliTaq DNA Polymerase (Applied Biosystems, Waltham, MA, USA) was used, and the reaction was set up in a Biometra TRIO cycler (Analytik Jena, Jena, Germany). The following temperature-time profile was used: An activation step of 95 °C for 10 min was followed by 45 cycles composed of denaturation steps (95 °C for 15 s), annealing steps (55 °C for 30 s), extension steps (72 °C for 90 s), and a final single cycle at 72 °C for 5 min.

In addition, real-time PCR was established using specific primers and probes (Table 2) [49]. The reaction was performed in a 20 µL reaction volume containing 100 ng DNA and the SensiFAST Probe No-ROX Kit (Meridian Bioscience Cincinnati, OH, USA). The cycling conditions used were initial denaturation for 5 min at 95 °C, followed by 45 amplification cycles at 95 °C for 15 s, annealing at 58 °C for 30 s, and extension at 72 °C for 30 s in a qTOWER3 G qPCR cycler (Analytik Jena, Jena, Germany).

### 2.6. Western Blot Analysis

Plasma samples were derived from the thawed blood probes. The Western blot was performed as described previously in detail using the recombinant R2 fragment of the gB protein of PCMV/PRV [19,51]. The samples were tested at a dilution of 1:150.

## 3. Results

### 3.1. Results of the PCR-Based Screening

DNA was isolated from the spleen and liver samples, and real-time PCRs were performed to screen for DNA viruses (Table 3). In addition, a conventional PCR and a real-time reverse transcriptase PCR were performed to test for PERV-A/C and HEV3, respectively (Table 3). PCMV/PRV was found in the spleen of 6 and in the liver of one of the 10 animals tested. PLHV-1 was found in all 10 animals, both in the spleen and the liver. The same result was obtained for PLHV-3. PCV3 was found in the spleen and the liver of one animal. PERV-C was found integrated in the genomes of all animals using a real-time PCR method and in 9 of 10 animals using conventional PCRs designated PCR1 and PCR4 [49]. The real-time testing for PERV-C was positive in all 10 animals. The animals were negative for the other viruses (PLHV-2, PPV-1, PCV2, PCV4, PERV-A/C, and HEV) in both organs (Table 3).

### 3.2. Results of the Western Blot-Based Screening

**Figure 1:**
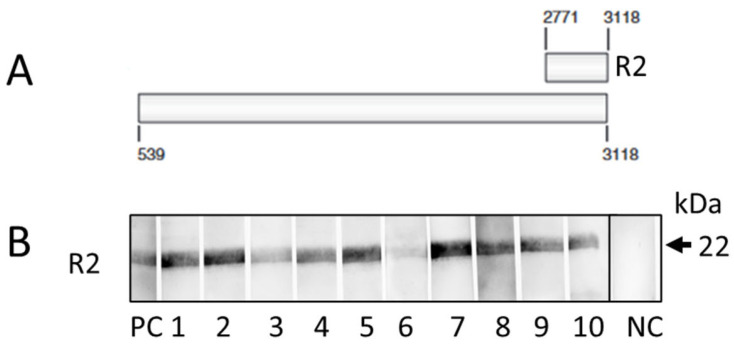
Western blot analysis of German slaughterhouse pigs. (**A**) schematic presentation of the glycoprotein gB of PCMV/PRV and localisation of the sequence corresponding to the recombinant protein R2 used as antigen (nucleotides according to Acc. No:AF268039) [19], (**B**) result of the Western blot of 10 animals using R2, PC, positive control, serum from a PCMV/PRV-positive pig, NC, serum from a PCMV/PRV-negative pig.

Plasma samples from the slaughterhouse pigs were screened for antibodies against PCMV/PRV by Western blotting using the recombinant R2 domain of the gB protein of PCMV/PRV as antigen. All animals were antibody positive, indicating that all animals were PCMV/PRV infected, either latently or an active infection (Table 1, Figure 1).

## 4. Discussion

To apply our methods developed to detect xenotransplantation-relevant porcine viruses, we screened 10 German slaughterhouse pigs. Surprisingly, the animals contained four DNA viruses: PCMV/PRV, PLHV-1, PLHV-3, and PCV3, although the animals were healthy and intended for consumption. There are hundreds of publications investigating the prevalence of pig viruses in slaughterhouse pigs worldwide. In most cases, they were screened for HEV [52,53,54], influenza virus [55,56], porcine reproductive and respiratory syndrome (PRRSV) [57], and Aujeszky’s disease virus [56]. For example, in the case of PRRSV, 74.3% of 1039 serum samples were seropositive, and 1.9% of the 1027 meat samples were positive by PCR in Canadian abattoirs. The virus was infectious when meat was ingested by uninfected pigs [57].

When we recently screened indigenous Greek black pigs using the same methods as described here, we found PCMV/PRV, PLHV-1, PLHV-2, PLHV-3, PCV2, and PCV3 [33] (Table 4). These animals were, among all the pigs we tested, the breed containing the highest number of pig viruses (Table 4). Since the animals were healthy and their meat was indented for consumption, it is possible that the animals have a natural resilience to virus infections, e.g., due to antiviral restriction factors [33]. Whether the German slaughterhouse pigs also have such restriction factors remains unclear.

All German slaughterhouse pigs had antibodies against PCMV/PRV, indicating that all were infected, despite the fact that virus DNA was not found in the liver and spleen of all animals by real-time PCR. This confirms previous results that PCMV/PRV, like all herpesviruses, establishes latency, during which it can no longer be detected using PCR methods [51]. This was the case for four animals when testing the DNA from the spleen and nine animals when testing the DNA from the liver. This is the only virus where differences between the detection in the liver and the detection in the spleen were observed; all other viruses were found in both organs concurrently. Obviously, at this stage of infection, more cells containing PCMV/PRV DNA remained in the spleen compared to the liver. Whether these cells are still expressing PCMV/PRV genes or even produce virus particles, or whether the DNA is derived from circulating latently infected cells, remains unknown.

As a rule, PCMV/PRV can be detected by PCR using nasal swabs only when an animal is freshly infected and suffers from rhinitis. PCMV/PRV can only be detected in the blood and organs of young animals by PCR only until approximately 19 weeks after infection [51]. In older animals, only the detection of virus-specific antibodies provides reliable data. For this, a Western blot analysis using recombinant proteins corresponding to the glycoprotein B (gB) of PCMV was developed [19,51]. PCMV gB is a transmembrane glycoprotein that plays a major role in fusion and adhesion when the virus enters cells. Since gB has good immunogenicity, as shown by us [19], it is well suited as antigen. Two fragments of the gB protein of PCMV/PRV, one N-terminal (R1) and one C-terminal (R2), were used in initial experiments, and since we could show that R2 was a better immunodominant region compared with R1 [19], we routinely used only R2. This was recently confirmed when ELISAs were performed using synthetic peptides derived from the same R2 region of gB [58]. Both methods, the Western blot assay using R2 and the ELISA using R2-derived peptides, gave comparable results [59]. We also produced the tegument proteins U54A (position 70307–72304, GenBank No. KF017583) and U54B (position 72345–73541) of PCMV/PRV and found antibodies against both proteins in infected pigs [60].

Antibodies against PCMV/PRV can also be detected in the blood of young animals. However, these antibodies are mostly from the infected mother sow, transmitted to the piglet by colostrum [51]. The transmission of PCMV/PRV to the first patient receiving a pig heart in Baltimore showed that screening for PCMV/PRV is not trivial. In this case, unfortunately, only nasal swab samples were taken from the donor animal and analyzed by PCR [3]. However, nasal swabs will only be positive when the animal is freshly infected and do not allow detection of latently infected animals [51,61].

Although PCMV/PRV is widely distributed in pigs worldwide and nearly all German slaughterhouse pigs are PCMV-positive [19] and this manuscript, the impact of PCMV/PRV on pig breeding seems to be low. PCMV/PRV infection is mostly acquired early in life, and infection results in seroconversion and life-long latent infection [62]. Fatalities were mainly observed in piglets less than 3 weeks old, and virus-infected sows are prone to abortion [62]. There are no antiviral drugs and no vaccines against PCMV/PRV. However, the virus can be easily removed from a pig population by early weaning [63]. Since PCMV/PRV is a roseolovirus closely related to human herpesviruses 6 and 7 (HHV6, 7) and only distantly related to the human cytomegalovirus (HCMV), all drugs effective against HCMV do not act against PCMV/PRV [64,65]. Various drugs against HCMV that were used to treat the first patient receiving a pig heart in Baltimore did not reduce the PCMV/PRV load in the patient’s blood [66].

When we screened for porcine lymphotropic herpesviruses, PLHV-1 and PLHV-3 were found in all animals. PLHV-1/3 are gammaherpesviruses widely distributed in pigs (up to 80% in single farms), but have not been associated with any pig disease (for review, see [67]). At present, there are no antiviral drugs or vaccines available. In contrast to PCMV/PRV, Caesarean delivery was not or only partially successful in eliminating these viruses [68,69]. PLHV was detected in all eight genetically modified pigs used for orthotopic heart transplantation; however, the virus was not transmitted to the baboon recipients [16].

When we screened for circoviruses, we found only one pig, which was infected with PCV3. The virus was found in both the liver and the spleen of animal 4. PCV1 is not pathogenic for pigs and was therefore not included into the screening. However, since we do not know whether it may be pathogenic for humans, it should be included in future testing of pigs intended for xenotransplantation. PCV2 induces an entire complex of porcine circovirus diseases (PCVD). There exist several vaccines against PCV2 that are able to prevent diseases, but in most cases, they do not prevent the transmission of the virus (for review, see [27]). PCV3 was discovered in 2016; it is common in domestic pigs and wild boars worldwide (for review, see [28]). Although PCV3 was also found in healthy pigs, there is clear evidence that PCV3 is pathogenic since virus clones were able to induce porcine dermatitis and nephropathy syndrome (PDNS) in specified pathogen-free animals [70]. PCV3 was also found in Greek pigs with erythema multiform [40] and in Göttingen minipigs with dippity pig syndrome [39]. In contrast to our findings in a German slaughterhouse, the prevalence of PCV2, PCV3, and PCV4 in slaughterhouses in one province in China was 56.8, 80, and 9.4%, respectively [71]. PCV3 was also found in pigs generated for xenotransplantation, which appeared clinically healthy, and the virus was transmitted in a few cases to baboons after transplantation of hearts from these pigs [72].

When we screened for HEV, all animals were negative. This is remarkable because in other slaughterhouses, higher amounts of positive animals were found. For example, HEV IgG was detected in sera from 167 pigs among 250 tested animals (67.6%), and HEV RNA was detected in 25 (11.0%) liver samples in Dutch slaughterhouses [54]. HEV RNA was detected in 6.3% and HEV IgG in 40% of 5033 serum samples from market-weight pigs at 25 slaughterhouses in 10 US states [53]. HEV is of great importance for xenotransplantation because it represents a proven zoonotic virus. It may induce diseases in humans, mainly moderate hepatitis and neurological syndromes (for review, see [73,74]). HEV was found in Göttingen minipigs, which are produced under specified pathogen-free conditions using real-time PCR and antibody detection by Western blot assays, and transplacental mother-to-piglet transmission was demonstrated [34].

Since all pigs carry PERV-A and PERV-B proviruses in their genomes, we only tested for PERV-C and recombinant PERV-A/C. PERV-C was found in all German slaughterhouse pigs as well as in all other pig breeds screened for it, with the exception of the Göttingen minipigs at Göttingen University (Table 4). It is interesting that in the German slaughterhouse pigs PERV-C was detected in all pigs using real-time PCR, but PERV-C was not detected in animal 3 using conventional PCR (Table 3). The PCR used was designated PCR4 by Kaulitz et al. [49]. PCR4 was shown to be highly reliable in the detection of PERV-C, comparable in reliability to PCR1 developed by Takeuchi et al. [75]. The reason for the discrepancy between PCR1 and PCR4 on the one hand and the real-time PCR on the other hand is under investigation [76]). Recombinants of PERV-A with PERV-C (PERV-A/C), which are characterized by higher virus titers and the ability to infect human cells, were not found in all investigated animals. This confirms previous findings that PERV-A/C recombinants are rare and found mainly in minipigs (for review, see [77]).

The methods used here and in previous investigations (Table 4) can not only be used for screening animals generated for xenotransplantation. They should also be used to screen all pigs used for biomedical research, because the presence of these viruses may interfere with the results of numerous biomedical experiments. Most importantly, these methods can also be used to eliminate these viruses from pig herds produced for consumption. This would improve the health of the animals, minimize losses in pig breeding, and lead to higher profits in meat production. Since xenotransplantation using pig cells, tissues, and organs may save and prolong the lives of patients but may also be associated with the transmission of porcine microorganisms to the recipient, eventually resulting in emerging infectious diseases, and since the methods developed here can also be used to reduce the virus load in pigs produced for consumption, the health of both the donor animals and the human recipients represents a special and sensitive case of the One Health concept [78].

## 5. Conclusions

PCR-based and immunological methods were used to screen German slaughterhouse pigs for eleven xenotransplantation-relevant viruses, including PERV. All pigs, with the exception of one, contained PERV-C in their genome. In addition, four DNA viruses were found. Together with previous screening of numerous other pig breeds, this indicates that these detection methods work well under field conditions. These diagnostic tests will be used to screen multiple genetically modified pigs designated for clinical trials of xenotransplantation. Furthermore, they can also be used to screen pigs used in biomedical research to prevent the viruses from influencing the resulting data. In pork production they may be used to reduce losses from these virus infections.

## Figures and Tables

**Table 1:** Sex and weight of the tested slaughterhouse pigs and results of the Western blot assay for antibodies against PCMV/PRV.

Animal	Sex	Weight (kg) *	PCMV/PRV
			Western Blot
1	male	110.9	+
2	male	108.1	+
3	male	97.6	+
4	female	97.6	+
5	female	99.1	+
6	male	93.8	+
7	female	108.2	+
8	male	109.3	+
9	female	95.6	+
10	female	94.7	+
Total	5 and 5		10/10

* after bleeding out; +, positive Western blot result.

**Table 2:** Oligonucleotides for the primers and probes used in this study.

Virus	Primer/Probe	Sequence 5′-3′	Reference
HEV3	JVHEV3-Fwd	GGT GGT TTC TGG GGT GAC	Jothikumar et al., 2006 [41]
	JVHEV3-Rev	AGG GGT TGG TTG GAT GAA	
	JVHEV3-Probe	6FAM-TGA TTC TCA GCC CTT CGC-BHQ	
PCMV/PRV	PCMV-Fwd	ACT TCG TCG CAG CTC ATC TGA	Mueller et al., 2002 [42]
	PCMV-Rev	GTT CTG GGA TTC CGA GGT TG	
	PCMV-Probe	6FAM-CAG GGC GGC GGT CGA GCT C-BHQ	
PLHV-1	PLHV-1 (1125)-Fwd	CTC ACC TCC AAA TAC AGC GA	Chmielewicz et al., 2003 [43]
	PLHV-1 (1125)-Rev	GCT TGA ATC GTG TGT TCC ATA G	
	PLHV-1 (1125)-Probe	6FAM-CTG GTC TAC TGA ATC GCC GCT AAC AG-TAMR	
PLHV-2	PLHV-2 (1155)-Fwd	GTC ACC TGC AAA TAC ACA GG	Chmielewicz et al., 2003 [43]
	PLHV-2 (1155)-Rev	GGC TTG AAT CGT ATG TTC CAT AT	
	PLHV-2 (1155)-Probe	6FAM-CTG GTC TAC TGA AGC GCT GCC AAT AG-TAMRA	
PLHV-3	PLHV-3 (210s)-Fwd	AAC AGC GCC AGA AAA AAA GG	McMahon et al., 2006 [44]
	PLHV-3 (210as)-Rev	GGA AAG GTA GAA GGT GAA CCA TAA AA	
	PLHV-3 (210)-Probe	6-FAM CCA AAG AGG AAA ATC-MGB	
PCV2	PCV2 (F2020)-Fwd	CTG AGT CTT TTT TAT CAC TTC GTA ATG GT	Chen et al., 2021 [45]
	PCV2 (F2020)-Rev	ACT GCG TTC GAA AAC AGT ATA TAC GA	
	PCV2 (F2020)-Probe	6FAM-TTA AGT GGG GGG TCT TTA AGA TTA AAT TCT CTG AAT TGT-BHQ2	
PCV3	PCV3-Fwd	AGT GCT CCC CAT TGA ACG	Palinski et al., 2017 [46]
	PCV3-Rev	ACA CAG CCG TTA CTT CAC	
	PCV3-Probe	6FAM-ACC CCA TGG CTC AAC ACA TAT GAC C-BHQ1	
PCV4	PCV4 (F2020)-Fwd	ATT ATT AAA CAG ACT TTA TTT GTG TCA TCA CTT	Chen et al., 2021 [45]
	PCV4 (F2020)-Rev	ACA GGG ATA ATG CGT AGT GAT CAC T	
	PCV4 (F2020)-Probe	6FAM-ATA CTA CAC TTG ATC TTA GCC AAA AGG CTC GTT GA-BHQ1	
PPV1	PPV1-Fwd	CAG AAT CAG CAA CCT CAC CA	Opriessnig et al., 2011 [47]
	PPV1-Rev	GCT GCT GGT GTG TAT GGA AG	
	PPV1-Probe	6FAM-TGC AAG CTT/ZEN/AAT GGT CGC ACT AGA CA-BHQ1	
pGAPDH	pGAPDH-Fwd	ACA TGG CCT CCA AGG AGT AAG A	Duvigneau et al., 2005 [48]
	pGAPDH-Rev	GAT CGA GTT GGG GCT GTG ACT	
	pGAPDH-Probe	HEX-CCA CCA ACC CCA GCA AGA G-BHQ1	
PERV-C, PCR1	PERV-envC-Fwd	GAT TAG AAC TGG AAG CCC CAA GTG CTC T	Kaulitz et al., 2013 [49]
	PERV-envC-Rev	TCT GAT CCA GAA GTT ATG TTA GAG GAT GGT	
PERV-C, PCR4	envC.2 for	GATTAGAACTGGAAGCCCCAAGTGCTCT	
	envC.2 rev	TCTGATCCAGAAGTTATGTTAGAGGATGGT	
PERV-C real-time PCR	PERV-C forward	CCCCAACCCAAGGACCAG	
	PERV-C reverse	AAGTTTTGCCCCCATTTTAGT	
	PERV-C probe	FAM-CTCTAACATAACTTCTGGATCAGACCC- BHQ1	
PERV-A/C	PERV-A env VRBF-Fwd	CCT ACC AGT TAT AAT CAA TTT AAT TAT GGC	Wood et al., 2004 [50]
	PERV-C env TMR-Rev	CTC AAA CCA CCC TTG AGT AGT TTC C	

Fwd = forward primer, Rev = reverse primer.

**Table 3:** Screening for pig viruses in spleen and liver of German slaughterhouse pigs (mean ct values).

SPLEEN													
Animal	PCMV /PRV	PLHV-1	PLHV-2	PLHV-3	PPV-1	PCV2	PCV3	PCV4	PERV-C	PERV-C		PERV-A/C	HEV
	Real-time PCR	Real-time PCR	Real-time PCR	Real-time PCR	Real-time PCR	Real-time PCR	Real-time PCR	Real-time PCR	Real-time PCR	PCR1	PCR4	PCR	Real-time RT-PCR
1	29.61	31.97	n.d.	27.41	n.d.	n.d.	n.d.	n.d.	24.57	+	+	-	n.d.
2	31.01	31.15	n.d.	28.24	n.d.	n.d.	n.d.	n.d.	21.67	+	+	-	n.d.
3	30.37	32.26	n.d.	27.77	n.d.	n.d.	n.d.	n.d.	26.43	-	-	-	n.d.
4	n.d.	31.21	n.d.	29.07	n.d.	n.d.	20.09	n.d.	25.11	+	+	-	n.d.
5	32.64	29.01	n.d.	35.62	n.d.	n.d.	n.d.	n.d.	25.78	+	+	-	n.d.
6	n.d.	31.75	n.d.	35.23	n.d.	n.d.	n.d.	n.d.	26.36	+	+	-	n.d.
7	34.10	28.56	n.d.	31.67	n.d.	n.d.	n.d.	n.d.	25.78	+	+	-	n.d.
8	n.d.	30.07	n.d.	28.96	n.d.	n.d.	n.d.	n.d.	23.49	+	+	-	n.d.
9	n.d.	33.95	n.d.	32.73	n.d.	n.d.	n.d.	n.d.	29.40	+	+	-	n.d.
10	36.49	32.57	n.d.	33.81	n.d.	n.d.	n.d.	n.d.	27.12	+	+	-	n.d.
Total	6/10	10/10	0/10	10/10	0/10	0/10	1/10	0/10	10/10	9/10	9/10	0/10	0/10
**LIVER**													
**Animal**	**PCMV** **/PRV**	**PLHV-1**	**PLHV-2**	**PLHV-3**	**PPV-1**	**PCV2**	**PCV3**	**PCV4**	**PERVC**	**PERV-C**		**PERV-A/C**	**HEV**
	**Real-time PCR**	**Real-time PCR**	**Real-time PCR**	**Real-time PCR**	**Real-time PCR**	**Real-time PCR**	**Real-time PCR**	**Real-time PCR**	**Real-time PCR**	**PCR1**	**PCR4**	**PCR**	**Real-time RT-PCR**
1	36.19	31.35	n.d.	31.75	n.d.	n.d.	n.d.	n.d.	28.66	+	+	-	n.d.
2	n.d.	31.68	n.d.	29.91	n.d.	n.d.	n.d.	n.d.	22.00	+	+	-	n.d.
3	n.d.	30.79	n.d.	33.00	n.d.	n.d.	n.d.	n.d.	31.05	-	-	-	n.d.
4	n.d.	30.12	n.d.	32.21	n.d.	n.d.	25.00	n.d.	26.46	+	+	-	n.d.
5	n.d.	27.64	n.d.	35.47	n.d.	n.d.	n.d.	n.d.	28.90	+	+	-	n.d.
6	n.d.	29.89	n.d.	34.92	n.d.	n.d.	n.d.	n.d.	26.19	+	+	-	n.d.
7	n.d.	27.46	n.d.	34.21	n.d.	n.d.	n.d.	n.d.	27.42	+	+	-	n.d.
8	n.d.	29.48	n.d.	31.81	n.d.	n.d.	n.d.	n.d.	23.05	+	+	-	n.d.
9	n.d.	34.84	n.d.	37.02	n.d.	n.d.	n.d.	n.d.	31.68	+	+	-	n.d.
10	n.d.	30.68	n.d.	35.50	n.d.	n.d.	n.d.	n.d.	33.57	+	+	-	n.d.
Total	1/10	10/10	010	10/10	0/10	0/10	1/10	0/10	10/10	9/10	9/10	o/10	0/10

n.d., not detected; ct values are shown for the real-time PCRs; +, positive result of a PCR; -, negative result of a PCR; the color indicates positive results, e.g., the presence of the virus.

**Table 4:** Summary of virus testing of different pig breeds.

Pig Breed	Virus Detection Method	PCMV/PRV		PLHV-1	PLHV-2	PLHV-3	PPV-1	PCV1	PCV2	PCV3	PCV4	PERV-C	PERV-A/C	HEV3		Reference
	Facility/ Institution	Real-Time PCR	Western Blot	Real-Time PCR	Real-Time PCR	Real-Time PCR	Real-Time PCR	Real-Time PCR	Real-Time PCR	Real-Time PCR	Real-Time PCR	Real-Time PCR, PCR	PCR	Real-Time RT-PCR	Western Blot	
Göttingen minipigs	Ellegaard Göttingen Minipigs A/S, Denmark	12/39 (30%)	8/67 (12%)	0/14 (0%)	n.t.	n.t.	n.t.	n.t.	3/21 (14%)	0/10 (0%)	n.t.	28/28 (100%)	3/13 (23%)	9/40 (22.5%)	2/22 (9%)	Morozov et al. [34,35], Plotzki et al. [19], Heinze et al. [36],
Göttingen minipigs	University Göttingen, Göttingen, Germany	0/10 (0%)	n.t.	2/11 (18%)	2/11 (18%)	2/11 (18%)	n.t.	n.t.	2/10 (20%)	0/10 (0%)	n.t.	0/10 (0%)	0/10 (0%)	0/10 (0%)	n.t.	Krüger et al. [24]
Göttingen minipigs with dippity pig syndrome	Ellegaard Göttingen Minipigs A/S, Denmark, Marshall BioResources, North Rose, New York	3/7 (42%)	n.t.	0/7 (0%)	0/7 (0%)	n.t.	n.t.	3/7 (42%)	0/7 (0%)	2/7 (29%)	0/7 (0%)	7/7 (100%)	0/1 (0%)	0/1 (9%)	n.t.	Jhelum et al. [39]
Aachen minipigs	Aachen Minipig, Heinsberg, Germany	5/18 (28%)	n.t.	0/18 (0%)	5/18 (28%)	3/18 (16%)	n.t.	n.t.	6/10 (60%)	n.t.	n.t.	13/13 (100%)	2/8 (25%)	12/18 (67%)	4/18 (22%)	Plotzki et al. [37]
Mini LEWE	University of Veterinary Medicine Hannover, Germany	0/10 (0%)	n.t.	0/10 (0%)	0/10 (0%)	0/10 (0%)	0/10 (0%)	0/10 (0%)	0/10 (0%)	0/10 (0%)	0/10 (0%)	10/10 (110%)	0/10 (0%)	0/10 (0%)	n.t.	Halecker et al. [38]
Indigenous Greek black pigs	Four farms in Greece	16/21 (76%)	11/11 (100%)	12/21 (57%)	15/21 (71%)	21/21 (100%)	0/21 (0%)	n.t.	21/21 (100%)	6/21 (29%)	0/21 (0%)	11/21 (52%)	0/21 (0%)	0/21 (0%)	n.t.	Jhelum et al. [33]
Greek pigs with erythema multiforme	Farm in Greece	0/5 (0%)	n.t.	5/5 (100%)	1/5 (20%)	4/5 (80%)	n.t.	0/5 (0%)	1/5 (20%)	1/5 (20%)	0/5 (0%)	5/5 (100%)	0/5 (0%)	n.t.	n.t.	Halecker et al. [40]
German slaughterhouse pigs	Slaughterhouse near Berlin, Germany	6/10 (60%)	10/10 (100%)	10/10 (100%)	0/10 (0%)	10/10 (100%)	0/10 (0%)	n.t.	0/10 (0%)	1/10 (10%)	0/10 (0%)	10/10 (100%)	0/10 (0%)	0/10 (0%)	n.t.	This manuscript

n.t., not tested; light brown color, virus present in some or all animals tested; light green color, virus absent in all animals tested.

## Data Availability

Data are contained within the article.
